# Linking the interaction of Salicylates and Jasmonates for stress resilience in plants

**DOI:** 10.1007/s44154-025-00250-9

**Published:** 2025-10-28

**Authors:** Ekta Pandey, Rinkee Kumari, Shahla Faizan, Saurabh Pandey

**Affiliations:** 1 Environmental Physiology Laboratory, Department of Botany, Aligarh Muslim University, Aligarh, U.P. 202002 India; 2 Department of Molecular Biology and Biotechnology, Indira Gandhi Agricultural University, Raipur, Chhattisgarh 492012 India

**Keywords:** Salicylate (SA), Jasmonate (JA), SA-JA crosstalk, Stress resilience, Biotic and abiotic stress, Molecular pathways

## Abstract

Plants are continuously exposed to environmental abiotic and biotic stressors that can significantly impact their growth, development, productivity, and lifespan. However, plants have developed exceptionally complex signaling pathways that enable their ability to sense, transduce, and respond to these diverse stress stimuli. Salicylates (SA) and jasmonates (JA) are two key phytohormones that significantly influence plant adaptation to environmental and biotic stressors, pivotal in enhancing stress resilience. The interaction and crosstalk between SA and JA signaling cascades are essential for orchestrating appropriate physiological and biochemical responses to biotic (e.g., pathogen attack, herbivory) and abiotic (e.g., oxidative stress, drought, temperature extremes, UV radiation, salinity, heavy metal toxicity) stresses. Salicylates are primarily recognized for being involved in systemic acquired resistance (SAR) against biotic stressors like pathogens. Conversely, jasmonates are well-documented in their function in defenses aimed at herbivorous insects and in mitigating the outcomes of abiotic conditions such as salinity and drought. However, the crosstalk between SAs and JAs is complex, involving both synergistic and antagonistic interactions that finely tune the natural defensive mechanism of the plant toward both biotic and abiotic stresses. This comprehensive review summarizes the most recent research on how SA and JA biosynthesis, signaling, and interactions govern diverse stress adaptive mechanisms in plants. It covers emerging evidence on the importance of SA-JA crosstalk in regulating physiological, biochemical, and molecular adaptations to combined biotic and abiotic stresses.

## Introduction

We need crop plants for our daily needs to provide us with food, fiber, and fuel (Waadt et al. 2022). In addition, crop plants contribute significantly to the diversity and sustainability of our planet's ecosystems. However, as sessile organisms, plants are constantly suffering from a myriad of abiotic and biotic stressors, like pest invasion, pathogenic diseases, drought, extremely high or low temperatures, heavy metal uptake, soil salinity, and nutrient deficiencies, which pose significant obstacles to crops' growth and development, eventually resulting in low crop yield (Bharti et al. 2021; Pandey and Singh 2024; Sinha et al. 2022).

Plant cells produce reactive oxygen species (ROS) such as (H_2_O_2_, OH•, O^2•–^ etc.) in limited amounts as byproducts of their metabolic processes. However, environmental factors, including drought, cold, high temperatures, Heavy metals, salt, and pathogens, can cause ROS overproduction and oxidative stress. Oxidative stress disrupts cellular homeostasis and damages proteins, lipids, and genetic material (RNA and DNA), affecting vital activities like photosynthesis and respiration and ultimately impacting yields from agriculture (Garcia-Caparros et al. 2021). Therefore, achieving crop resilience to environmental stressors is imperative for the long-duration sustainability of agricultural systems and environmental conservation. Understanding the stress resilience mechanisms enabling plants to withstand adverse conditions ensures their survival and productivity. Consequently, knowledge of complicated networks of signaling pathways involved in stress response is essential for developing resilient crop varieties.

Plant growth regulators (PGRs) are integral to these networks and signaling pathways and act as stress sensors (El Sabagh et al. 2022) and successfully produce climate-resilient plants with high crop yields. Recently, PGRs have emerged as an eco-friendly option to improve biotic and abiotic stress resilience in horticulture crops. PGRs are chemical growth regulators secreted in small quantities and regulate agricultural crop growth, development, and environmental response. They function as chemical messengers to link cellular processes, coordinate signaling pathways, and regulate physiological responses to external environmental stimuli (Madaan et al. 2022; Bhatt et al. 2020). Synergistic or antagonistic biosynthesis of PGRs and signaling control are vital for plant survival (Khan et al. 2020). Among all PGRs, SA and JA have emerged as essential subjects in modulating stress responses and increasing the resistance to pathogenic and climatic stress. SA and JA play an extensive role in growth and development, influencing germination, root and tuber growth, embryogenesis, fruit and flower development, leaf senescence, and stomatal opening (Çetinbaş-Genç and Vardar 2021). Notably, SA has been identified to promote the gene expression associated with heat shock proteins (HSPs), chaperones, antioxidant enzymes, and secondary metabolites, including the notable examples of sinapyl alcohol dehydrogenase (SAD) and cytochrome P450. Furthermore, SA governs the activation of mitogen-activated protein kinases (*MAPKs*), cascades and orchestrates the expression and activation of pathways such as PAMPs-triggered immunity (PTI), effector-triggered immunity (ETI), and the non-expression of *pathogenesis-related genes 1 (NPR1)* (Ramakrishnan and Zhou 2022; Ullah et al. 2023). Research on Arabidopsis mutants affecting SA synthesis and signaling mechanisms concluded that SA is a vital defense chemical against biotrophs (Pluharova et al. 2019). JAs also control gene activation in response to abiotic and biotic stressors by promoting defensive mechanisms. The PGRs SA, JA, abscisic acid (ABA), cytokinin (CK), ethylene (ET), and auxins act together to control plant metabolism and development under environmental stress. In which the antagonistic pathways of JA and SA hormones play a prominent role in plant stress resistance. JA-SA crosstalk is mediated via multiple genes, including *MYC2*, *MAPKs*, *TGAs* (*TF family*), *PDF 1.2* (*plant defensin 1.2*), *NPR1*, *WRKY62* & *70* (*transcription factor 62&70*), *ERF1*(*ethylene-responsive factor 1*), *ORA59* (*oxidative stress response 59*), *GRX480* (*glutaredoxin 480*), and *JAZs* (*Jasmonate ZIM-domain*) (Vos et al. 2013a; Khan et al. 2023). All land plant progenitors have the ortholog *NPR1*, indicating that JA-SA crosstalk occurs within all plants (de Vries et al. 2018).

This review explores the exciting connection between SA and JA as plant growth regulators and stress tolerance molecules. By studying the complex signaling pathways and molecular mechanisms that govern SA-JA crosstalk, we hope to understand how these molecules coordinate stress resilience in plants and develop new crop productivity and sustainability strategies.

## SA and JA: important PGRs in plant growth regulation

PGRs, also known as growth controllers/plant hormones, are crucial because they control various developmental activities. These processes include flowering, fruit development, stem elongation, and seed germination. Plant hormones [including auxins, CKs, SA, GAs, ET, ABA, JA, and brassinosteroids (BRs)], polyamines (PAs a group of PGRs with aliphatic nitrogen structure), and nitric oxide (NO, a gaseous molecule), are among the prominent PGRs that have attracted the attention of agronomists and physiologists as a sustainable medium for inducing tolerance in plants that have been subjected to abiotic and biotic stresses (Pal et al. 2023). PGRs regulate plant growth through a general mechanism involving multiple types of PGRs, in which auxins promote cell elongation and differentiation, root initiation, and apical dominance and regulate plant growth by influencing stem and root cell division and elongation, which affects plant height and root development. Cell division and lateral bud expansion depend on CKs to promote plant growth and delay senescence. They control apical dominance, shoot, and leaf growth by interacting with auxins (Jing and Strader 2019). GAs stimulate stem elongation, seed germination, and flowering. GA regulates plant growth with auxins and CKs, particularly fruit development and plant height. ABA controls plant development by influencing seed dormancy, germination, and responses to abiotic stresses, such as metal, drought, heat, and salt. ET maintains plant development by affecting fruit maturity, leaf and petal abscission, and abiotic and biotic stresses. BR regulates plant growth by controlling cell proliferation, vascular differentiation, and physiological stress responses. Spermidine, spermine, and Putrescine are crop plants' principal low-molecular-weight polyamines (PAs). They control multiple biological processes, such as root growth, cell division, flowering, fruiting, transcription, embryo development, organogenesis, morphogenesis, and leaf senescence (Killiny and Nehela 2020).

SA and JA are the most often utilized growth regulators to alleviate abiotic stress or hazardous pathogenic microbes. Plants synthesize these internal growth-regulating compounds and usually trigger antioxidant mechanisms such as peroxidase, superoxide radical, the AsA-GSH pathway, and NADPH-oxidase (Sirhindi et al. 2020). This process leads to the accumulation of amino acids (tryptophan, arginine, isoleucine, methionine, and proline), phenols, soluble sugars, alkaloids, carotenoids/chlorophylls (Cirak et al. 2020), as well as the modulation of stomatal opening and closing movements (Miclea et al. 2020). Cellular responses often necessitate the activation of genes associated with SA, such as *PR 1, 3& 5 (Pathogenesis-Related Protein 1, 3&5), NPR1*, and *PAL (Phenylalanine Ammonia-Lyase)*, as well as genes linked to JA, including *JAZ, PDF 1.2, THI2.1* (*Thionin 2.1*), *AOS1, AOC, LOX2* (*Lipoxygenase 2*), and *COI1*. Furthermore, these responses involve intricate reactions with other plant hormones (CK, ABA, BR, GA, ET, and IAA) and cross-talk with transcription factors (TFs) such as *bHLH148* and *MYC2* (Wang et al. 2020; Zhao et al. 2013). This section will explore the biosynthesis and role of SA and JA in plant growth and developmental processes.

### SA: biosynthesis and role in plant growth regulations

**Fig. 1:**
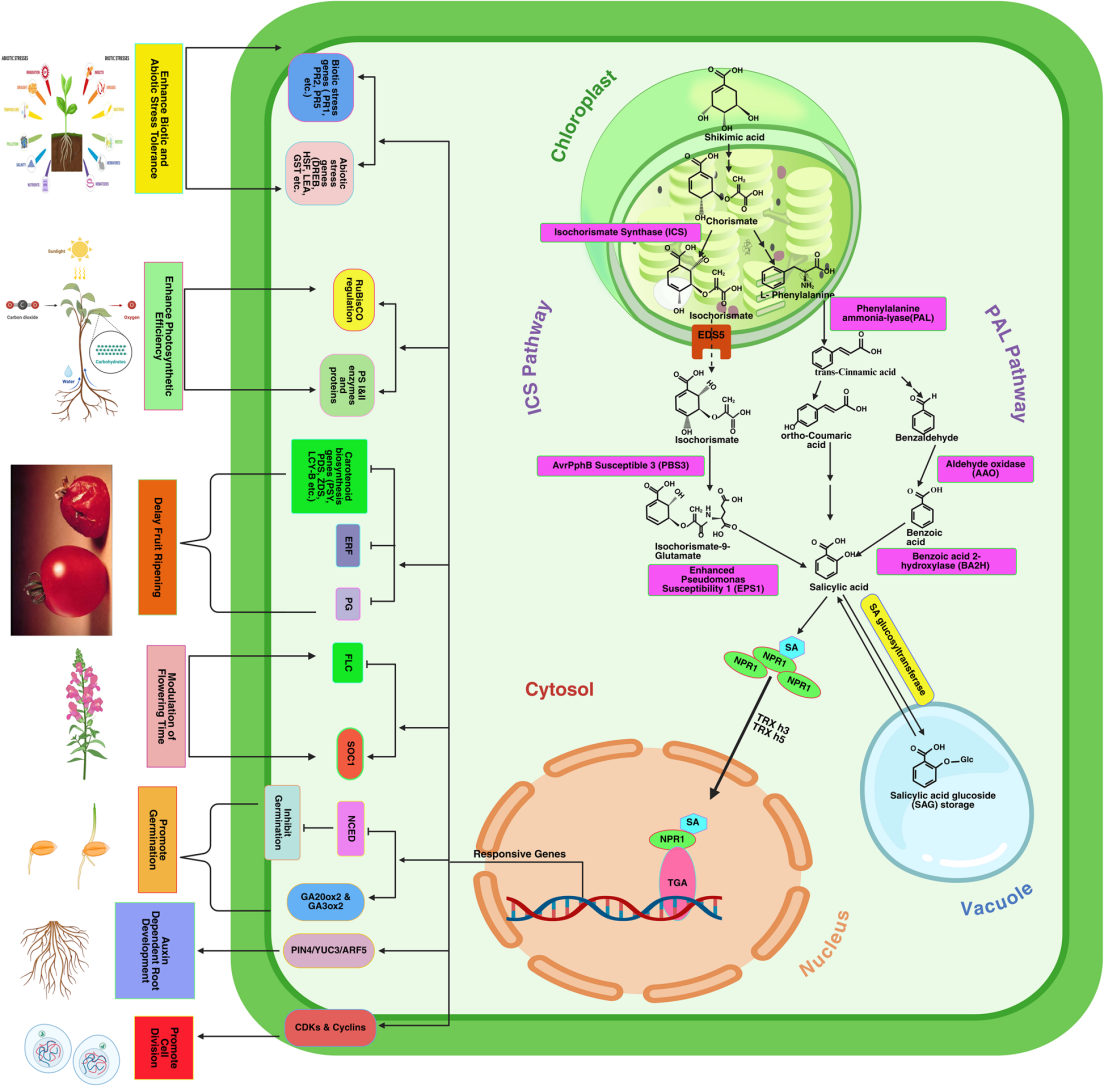
Biosynthetic pathway and response mechanism of salicylic acid to plant growth, development, and stress. Plants synthesize salicylic acid (SA) via the phenylalanine ammonia-lyase (PAL) and isochorismate (IC) pathways, with shikimic acid as a precursor to chorismic acid. Enzymes such as ICS, PBS3, EPS1, PAL, AAO, and BA2H facilitate the transformation of chorismate into salicylic acid. Glucosyltransferases synthesize salicylic acid glucosides (SAG and SGE) in vacuoles as a storage molecule. SA plays a crucial role in growth regulation by monomerizing NPR1, which enters the nucleus via TRX h3/h5 and interacts with TGAs at SA-responsive promoters. This activates genes and TFs involved in cell division (CdKs, cyclins), root development (PIN4/YUC3/ARF5), seed germination (activates GA20ox2, GA3ox2; inhibits NCED), flowering (activates SOC1; suppresses FLC), fruiting (inhibits ERF, PG, carotenoid genes), photosynthesis (regulates PSI, PSII, RuBisCo), and defense (activates PR1, 2, 5, HSF, DREB) (Created with BioResnder.com)

SA is a ubiquitous small phenolic molecule in green algae (Chlorophyta and charophyte) and terrestrial plants. It serves as a widespread PGR in the green lineage, indicating that the most recent ancestral lineage of Viridiplantae probably synthesized SA and its derivative. While certain bacteria can manufacture SA from isochorismate using pyruvate lyase directly, green plants rely on two biosynthetic routes to produce SA via chorismate: namely, the PAL route and the isochorismate synthase (ICS) route, as illustrated in Fig. 1. Plants exhibiting elevated SA levels, a result of heightened activity of enzymes within the SA biosynthesis pathway, particularly PAL and ICS, demonstrate enhanced resilience to environmental challenges. It is widely understood that numerous biotic and abiotic stress factors can impact these enzymes, which serve as critical regulators of SA functions. Researchers have discovered that ICS is crucial in producing SA in Arabidopsis during plant defense (Hu et al. 2022).

Exogenous SA's growth-inducing effects are species- and concentration-dependent. Regarding plant and organ development, varying SA concentrations have opposite impacts on different species. For example, Kaur et al. (2022) established that applying 0.5 mm SA improved the dry weight of chickpea shoots, roots, and nodules and increased the count of flowers and pods. Correspondingly, studies by Khan et al. (2013, 2014) revealed that using 0.5 mm SA significantly augmented the photosynthetic rate and growth progression in wheat and mungbean. One of SAs functions is to block the enzyme catalase. Reducing catalase enzyme activity increases hydrogen peroxide, which may help certain seeds germinate better (Anaya et al. 2018). SA also improves *mes7* mutant seed germination under various circumstances (Gao et al. 2021). Extensive research on SA signaling in plant immunological responses has pinpointed *NPR1* as an essential regulator of this mechanism. Beyond its involvement in growth regulation mediated by SA, NPR1 is vital in regulating cell proliferation and division. Research conducted by Vanacker et al. (2001) and Li et al. (2022) suggests that the decline in cell number and augmented DNA amount observed in Arabidopsis *npr1-1* mutant leaves indicate that *NPR1* stimulates cell division and simultaneously inhibits endoreduplication. Fujikura et al. (2020) discovered that *xs2* mutant cells were smaller than wild-type cells due to poor cell growth and excessive SA accumulation. Surprisingly, the *xs2 npr1* double mutant brought back the notable cell size deficiency observed in the *xs2* mutant. Also, the SA-overaccumulating Arabidopsis mutant *cad1* exhibited an increase in QC cell division that was restored by mutating *SID2* or *NPR1*. This suggests that the *cad1* mutant's and *xs2* mutant enhanced QC cell division and cell growth via an *NPR1*-dependent SA signaling mechanism (Wang et al. 2021b).

Additionally, SA has a small function in the seed germination process by suppressing the activation of the *NCED* (*9-cis-epoxy carotenoid dioxygenase*) gene, which is responsible for ABA biosynthesis and germination inhibition. Exogenous SA can limit the transcription of the ABA biosynthetic gene *NCED5* by simultaneously increasing the activity of the GA catabolic genes *GA20ox1* (*Gibberellin 20-oxidase 1*) and *GA3ox2* (*Gibberellin 3-oxidase 2*), which are accountable for GA production (Liu et al. 2022). SA application can potentially activate the DNA Damage Response (DDR) by influencing the signaling pathways associated with DNA damage. Activating protein kinases like *ATM* (*Ataxia Telangiectasia Mutated*) and *ATR* (*ATM and Rad3-related*) is an integral part of this process because these kinases detect DNA damage and coordinate the repair of damaged DNA (Yan et al. 2013). Table 1 and Fig. 1 summarize SA's role in regulating and activating different TFs in the main growth processes in plants.

**Table 1:** SAs and JAs crosstalk in plant growth regulation

S.No	Plant Growth Phenomenon	SA Role	JA Role	Crosstalk Interaction	References
1.	Seed Germination	Usually, it reduces germination to focus on defense mechanisms. In certain situations, it boosts germination through enhanced *GA-responsive genes (GA20ox2 &GA3ox2)* expression.	JA increases alpha-amylase production, hydrolyzing starch into glucose, giving the embryo energy and breaking dormancy. JA also induces ROS production, which weakens the seed coat and promotes cell proliferation.	JA and SA influence gene expression but activate separate genes or signals via various pathways that may overlap or diverge. JA may trigger genes that break dormancy, whereas SA may activate a pathway that strengthens it. JA might mitigate SA's germination inhibitory effects in stress-free settings.	(Liu et al. 2022; Sybilska and Daszkowska-Golec 2023; Bailly 2019)
2.	Root Development	-Promotes root elongation and branching at low concentrations. - SA modulates root apical meristem (RAM) auxin-dependent activities via affecting auxin transporters such as PIN proteins and auxin response factors (*ARFs*) activity.	- Involved in root growth inhibition under stress conditions, promoting defensive secondary root formation. JA master regulator *MYC2* minimizes root meristematic cell activity and inhibits primary root development by suppressing *PLETHORA genes (PLT1 and PLT2)*.	- JA and SA may exert a detrimental effect on primary root elongation. It can have a synergistic impact on the promotion of lateral root development. JA and SA have distinct but interdependent root hair development and growth functions.	(Bagautdinova et al. 2022; Pasternak et al. 2019; Ghorbel et al. 2021; Lakehal and Bellini 2019)
3.	Flowering	- Modulates flowering time regulating genes such as *FLOWERING LOCUS C (FLC)* and *SUPPRESSOR OF OVEREXPRESSION OF CO 1 (SOC1)*. - Indirectly regulates *CONSTANS (CO)*, a crucial gene in photoperiodic flowering control.	- Regulates the transcription of FLOWERING LOCUS T (FT) genes, which are critical for the transition from vegetative growth to flowering. - JA modulates genes assisting in petal, stamen, and pistil development, ensuring proper reproductive structure formation. - JA produces volatile organic compounds (VOCs), which attract pollinators.	- SA and JA can individually regulate the expression of critical flowering time genes such as *FLOWERING LOCUS C (FLC), CONSTANS (CO),* and *SUPPRESSOR OF OVEREXPRESSION OF CO 1 (SOC1)*. - SA-JA antagonism allows plants to fine-tune strategies and prioritize either growth/reproduction or defense according to environmental conditions. - Under stress, SA and JA's antagonistic interaction may delay flowering and the reproductive phase.	(Luo et al. 2022; Zhao et al. 2022; Verma et al. 2016)
4.	Fruit Development	- Less active participation in fruit development; more attention to pathogen defense during fruiting - Sometimes, SA can delay the fruit ripening by influencing the expression of genes like *POLYGALACTURONASE (PG), ETHYLENE RESPONSE FACTOR (ERF),* and *CAROTENOID BIOSYNTHESIS genes*.	- JA typically acts with ethylene, a fruit-ripening hormone. It regulates ethylene biosynthesis via stimulating ethylene genes. - JA regulates fruit color by altering anthocyanin and carotenoid accumulation. It affects enzymes that change cell wall characteristics, affecting ripening fruit texture.	- SA and JA can have antagonistic interactions during the initial stages of fruit development. - Interaction adjusts the balance between defense and development during fruit maturation and ripening	(Xu et al. 2023; Fenn and Giovannoni 2021; Forlani et al. 2019)
5.	Leaf Senescence	- Participate in the leaf senescence by activating *MAPKs, MAKs*, and *WKRY TF*, which inhibits *REVOLUTA*, a key regulator of green leaf development	- JA plays a crucial role in leaf senescence by interacting with *MYC5* and* SAG29*	- Both work synergistically by interacting with *SAGs* (Senescence Associated GENES)	(Bresson et al. 2022)
6.	Photosynthesis	- SA regulates chlorophyll and carotenoid pigment genes. -SA influences Photosystem I and II enzymes and proteins in the photosynthetic electron transport chain - SA regulates RuBisCO, a key carbon fixation enzyme.	- JA Affects photosynthetic efficiency under stress conditions by regulating leaf senescence and closure of stomata. - JA influences photosynthetic electron transport chain genes such as *PsbA, PsbD*, and *PetE*. - JA modulates stomatal development and function genes (e.g., *SPEECHLESS, MUTE, FAMA*).	- SA-JA interactions may influence the allocation of resources towards either photosynthetic efficiency or defensive compounds - *WRKY* transcription factors are key in coordinating SA and JA instructions to coordinate photosynthesis-related gene expression. - *ERF* transcription factor. Impacts photosynthetic processes via SA, JA, and ethylene signaling.	(Janda et al. 2014; Balfagón et al. 2019; Falquetto-Gomes et al. 2023; Zhang et al. 2020; Caarls et al. 2015)

### JAs: biosynthesis and role in plant growth regulation

**Fig. 2:**
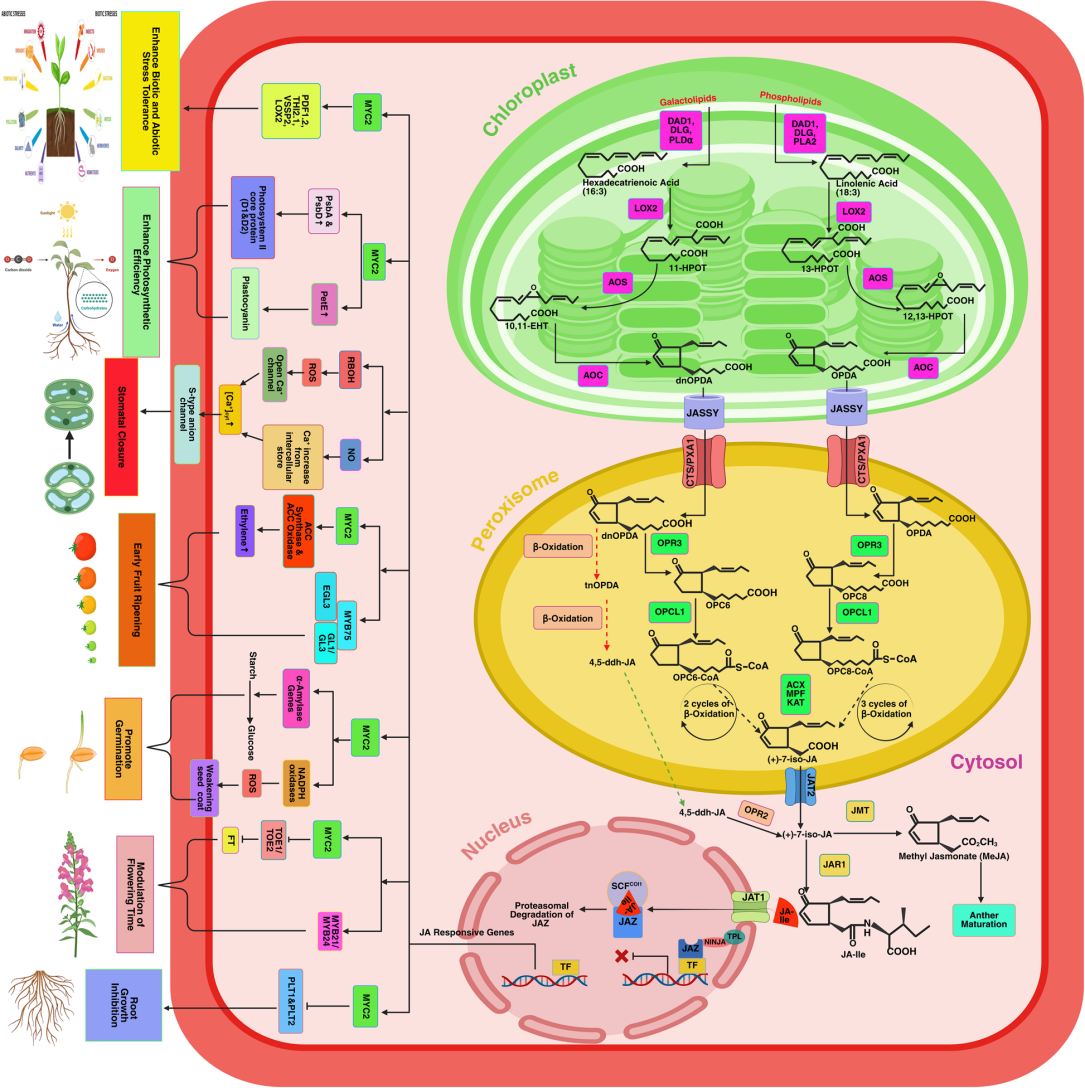
Jasmonic acid biosynthetic pathway and signaling in plant growth, development, and stress response. In response to developmental or defense signals, JA-Ile is rapidly synthesized and recognized by the F-box protein COI1, which recruits JAZ repressor proteins for ubiquitination and destruction via the 26S proteasome. This derepresses the NINJA-TPL complex, allowing transcription factors like MYC2 to regulate diverse processes: Inhibits root growth by suppressing PLT1/2, Modulates flowering time by inhibiting TOE1/2, Promotes seed germination by activating α-amylase and NADPH oxidases, Enhances photosynthesis and defense (PsbA, PsbD, PetE, PDF1.2, THI2.1, VSP2, LOX2), early fruiting (ACC synthase and oxidase). JA also regulate Regulates early fruit development and flowering by activating MYB75, EGL3, GL1/3, MYB21/24 and promotes stomatal closure by modulating NO, ROS, and Ca2 + .( +)-7-iso-JA convert into MeJA by jasmonate methyl transferase enzyme which promote the anther maturation (Created with BioRender.com)

JA, with jasmonic acid isoleucine (JA-Ile) and methyl jasmonate (MeJA) (metabolic derivatives of JA), represents a class of lipid-based phytohormones prevalent in higher plants. JA influences seed germination, seedling growth, root development, tuber formation, leaf senescence, leaf movement, fruit maturation, chlorophyll degradation, and secondary metabolite production. The discovery of jasmonate can be traced back to *Jasminum grandiflorum*, where it was first identified as a methyl ester. JA belongs to the family of cyclopentane fatty acids, which are synthesized within plant cells through the octadecanoid pathway. This pathway involves enzymes such as allene oxide synthase, lipoxygenase, allene oxide cyclase, and oxophytodienoic acid reductase (AOS, LOX, AOC & OPR), which work together to convert α-linolenic acid, a critical fatty acid in plasma membranes, into JA (Fig. 2). The biosynthesis initiation and termination sites are present in the chloroplast membranes and the peroxisome. Afterward, the end product ( +)-7-iso-JA is subsequently transferred to the cytoplasm, where it undergoes additional modification by jasmonate methyl transferase (JMT) and jasmonate-amido synthetase (JAR1) to produce MeJA or JA-Ile derivative (Ruan et al. 2019; Aslam et al. 2021). Transferred into the cellular nucleus, the metabolized JA-Ile activates several crucial TFs, including *MYC2*, to facilitate the expression of JA-responsive genes downstream, using a "relief of repression" model. The regulatory elements of the JA signaling cascade are *MYC* proteins.

The *MYC* family is abundant in bryophytes and angiospermae, comprising an arm of the basic helix-loop-helix (bHLH) superfamily (Peñuelas et al. 2019). *MYC2* reduced auxin biosynthesis to limit leaf vein growth. *MYC2* adversely regulates leaf vein formation. JA signaling causes *MYC2* to bind to the G-box promoter of *PLETHORA (PLT)*, reducing its target genes. JA influences Arabidopsis primary root growth inhibition. JA hinders root meristem proliferation and changes stem cell niche cellular structure. For example, *MYC2* directly suppresses *PLT1* and *PLT2* after JA treatment, emphasizing that *MYC2*-mediated *PLT* repression is required for JA-induced root meristem growth and stem cell niche preservation (Chen et al. 2011). *MYC2* also influences seed size, weight, and storage protein development. For example, *AtMYC2* positively correlates with seed storage rate at different stages. JA controls stamen filament elongation, anther dehiscence, and pollen vitality. JA non-functionality often causes acute male sterility. Mutants *opr3*, *aos* of JA biosynthesis, and *COI1* (*coi1-1*) receptor of JA in Arabidopsis produce defective anther and pollens, which cause male sterility. JA also represses the vegetative-reproductive phase transition. In Arabidopsis, the JA pathway, involving the *COI1-JAZ/TOE-FT* module, acts as a negative regulator of flowering. A *coi1-2* mutant, *JAZ1∆3A* genetically engineered plant, and *JAZ9* overexpression plants exhibited early flower blooming. *FLOWERING LOCUS T* transcription is repressed by *JAZ* proteins and *APETALA2 (AP2)* family TFs *TOE1* and *TOE2*. *SlJAZ2* controls tomato morphology and flowering. *SlJAZ2*-overexpressing plants had faster leaf development, reduced total plant height and internode length, faster lateral bud emergence, and earlier flowering transition from a vegetative state. MeJA triggers stomata closure in many plant species, while the exact mechanism of the MeJA signaling pathway remains uncertain. Recently, we discovered that the Arabidopsis *calcium-dependent protein kinase 6 (CPK6)* plays a role as a regulatory protein in the signaling pathway of guard cells responding to MeJA. This finding has given us a fresh understanding of how cytosolic calcium levels influence MeJA signaling (Munemasa et al. 2011). Suhita et al. (2004) discovered that the loss of two Arabidopsis NAD(P)H oxidases, *Atrbohf* and *Atrbohd*, fails MeJA-induced stomatal closure and the formation of ROS. JA also regulates plant growth by increasing resistance to pathogen attacks and environmental stress. Plants infested by the *Pieris rapae* (small cabbage white butterfly) elevate JA and ( +)-7-iso-jasmonoyl-L-isoleucine to equivalent levels in damaged leaves, thereby inducing herbivore resistance through the activation of *MYC2* and *VSP1*(Vos et al. 2013b). JA also participates in the activation of *PHYTOALEXIN DEFICIENT 4* (*PAD4*), *ENHANCED DISEASE SUSCEPTIBILITY 1* (*EDS1*), and *SENESCENCEASSOCIATED GENE 101* (*SAG101*) genes, which provide plant pathogens resistance (Reinbothe et al. 2009). Table 1 and Fig. 2 show the role of JA in activating multiple TFs, which directly or indirectly regulate different stages of plant growth.

### SAs and JAs crosstalk in plant growth regulation

**Fig. 3:**
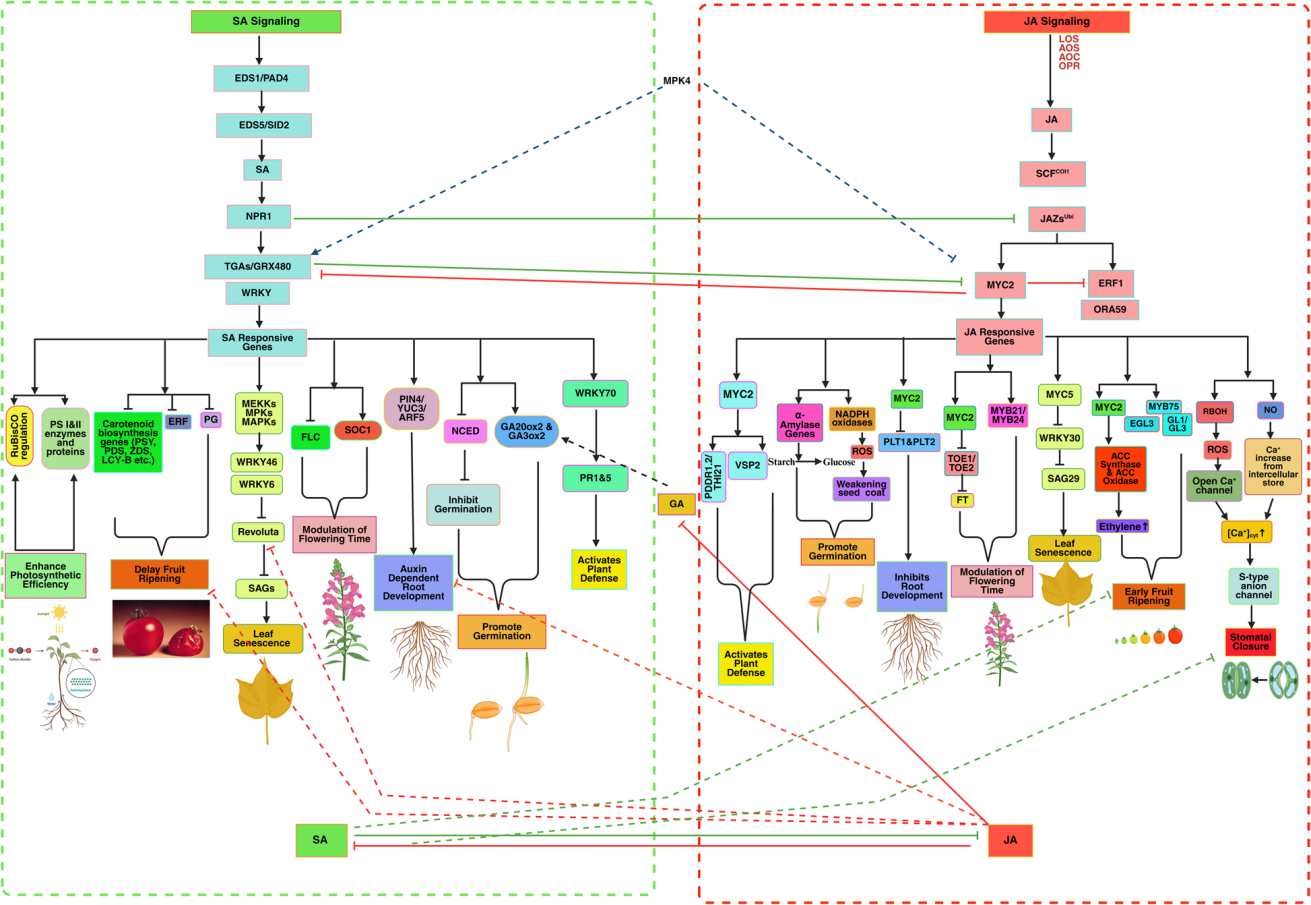
SA and JA-mediated crosstalk in plant growth, development, and stress response. The SA and JA signaling pathways have antagonistic and synergistic interactions that influence plant growth and stress responses.TGAs/GRX480 and MYC2 mediate reciprocal inhibition of the SA and JA pathways. MYC4 interacts negatively with JA-responsive MYC2 but positively with SA-responsive WRKY.SA stimulates seed germination by activating GA20ox2/GA3ox2, but JA reduces it by inhibiting GA.Root development: SA stimulates auxin-dependent PIN4/YUC3/ARF5, but JA inhibits auxin-dependent root initiation. Fruit ripening: JA stimulates ERF, boosting ripening, while SA inhibits JA and prevents early ripening. Stomatal closure: SA and JA synergistically inhibit the REVOLUTA gene (Created with BioRender.com)

As we've already discussed, JA and SA are important phytohormones that influence plant growth and development by changing the gene expression levels involved in different plant growth processes (Figs. 1 and 2). However, the interaction of SA and JA regulates plant growth by providing immunity from severe pathogen attacks and environmental stresses (Fig. 3 and Table 1). SA- and JA-mediated defensive signaling routes interplay synergistically and antagonistically (Table 1). Applying minimal concentrations of JA and SA induces synergistic expression of the SA-responsive gene *PR1* and the JA target gene *PDF1.2*. In contrast, higher levels induce antagonistic responses in both hormone-responsive genes (Mur et al. 2006).

Given that phenotypically heterogeneous adversaries are sensitive to various defensive mechanisms, reciprocated antagonism between the SA and JA crosstalk has been extensively documented, and it is vital in influencing the result of plant-pathogen interactions. This approach also constitutes an economic strategy as it influences the results of such interactions (Thaler et al. 2012). For instance, certain strains of the biotrophic leaf pathogen *Pseudomonas syringae* enhance their pathogenicity by deploying the JA-mimicking toxin coronatine, which inhibits SA-mediated immune defenses against this pathogen. As a result of the development of techniques to inhibit the virulence-promoting effect of coronatine, several plant hosts have inverted the established order (Geng et al. 2014; Spoel and Dong 2024). While suppressing the JA, *NPR1* SA responsive gene in both regional and systemic tissues, SA may have conflicting effects as a cell death against and a cell survival signal to give durable defense across the plant against various diseases. Therefore, SA may also play a role in cell death prevention (Zavaliev et al. 2020). JA–SA crosstalk is mediated by several genes, including *MYC2*, *REV* (*REVOLUTA*, a member of the class III homeodomain leucine zipper (HD-ZIPIII) TF family), *TGAs* (TF family), *MAPK, PDF 1.2, NPR1, WRKY62, WRKY30, WRKY53, WRKY70, ERF1, TGA, GRX480 (glutaredoxin 480), SAGs, ORA59 (octadecanoid-responsive Arabidopsis AP2/ERF 59), AGO10, RBOHS* and *JAZs* (Shigenaga et al. 2017; Li et al. 2019; Stroud et al. 2022). The homolog *NPR1* was found in the progenitor of all terrestrial plants, supporting JA–SA crosstalk in all plant species (Monte 2023). JA also affects *MYC2's* interaction with three *NAC* (TF family) genes *(ANAC072, ANAC055, and ANAC019)* to prevent SA buildup and control SA biosynthesis genes (Allu et al. 2016). MPK4 positively modulates *GRX480* (SA signaling route) but negatively modulates *MYC2* (JA signaling pathway). *GRX480* binds to *TGAs*, influencing *PR1* expression. *GRX* genes inhibit *ORA59*, a JA response gene (Choudhary et al. 2021). JA and SA help the plant to withstand different conditions by modulating the plant’s growth activities, such as both increasing the accumulation of ROS, NO, and intracellular Ca^2+^ concentration, which ultimately induce the activation of *MAP kinases* cascades and Redox status of chloroplast by SA induced *GRX480* expression. JA-SA accumulated Ca^2+^ ion increases the cell elongation, redox status of chloroplast helps the plant to maintain growth and development, *MAPK* enhances the tolerance level of plant to environmental stresses while NO causes stomatal closure.

### SAs and JAs crosstalk in biotic stress resilience

**Fig. 4:**
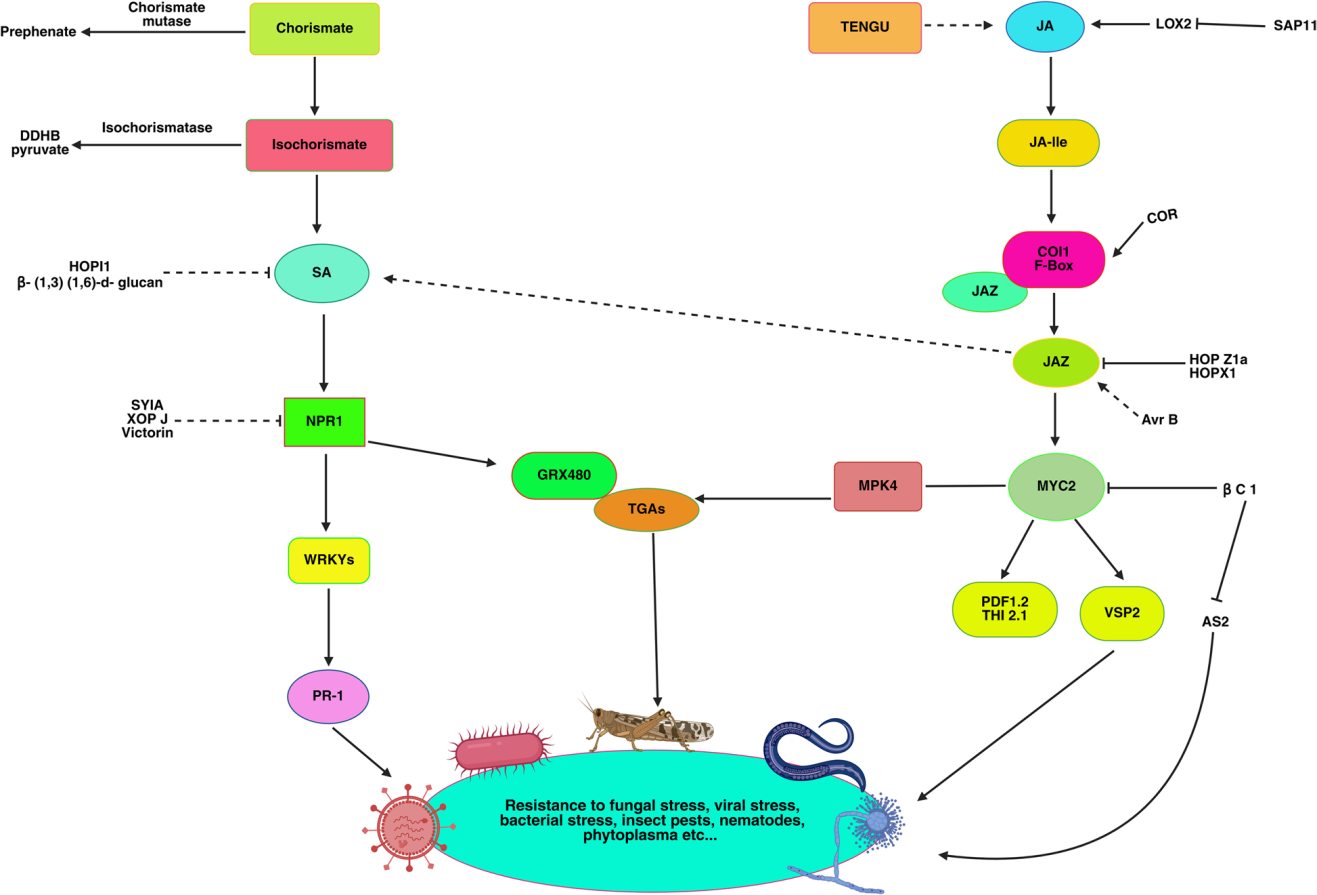
JA and SA signaling pathways complex crosstalk in biotic stress. In the necrotrophic stage of necrotrophic or hemi-biotrophic pathogens, SA initiates early expression of defense-related genes. In contrast, JA initiates late expression of defense-related genes against pathogen-attacked plants to provide plant resistance. The diagram illustrates the mechanism underlying increased stress tolerance in plants. It depicts the signaling pathways and regulatory processes activated in response to biotic stress. The central part of the diagram shows the plant, with various stress factors (pest, viral, bacterial, fungal, etc.) acting as inputs. These stresses trigger the activation of the SA (salicylic acid) and JA (jasmonic acid) pathways, which regulate the expression of key transcription factors and regulatory proteins, such as NPR1, WRKY VSP2, PR1, etc. Activating these transcriptional regulators leads to the induction of downstream stress-responsive genes, including those involved in producing protective compounds like MPK4 MYC2, JAZ, PDF1.2, THI 2.1, MYC, etc. The cumulative effect of these stress-responsive pathways and gene expression changes results in increased tolerance to various stresses, including fungal stress, viral stress, bacterial stress, insect pests, nematodes, and phytoplasma, as depicted by the "Increased stress Tolerance" outcome in the diagram (Created with BioRender.com)

SAs and JAs crosstalk in biotic stress resilience have been studied by various researchers, and their pathways are deduced by these studies, as presented in Fig. 4 (Table 2). JA generally regulates plant necrotrophic disease resistance. Meanwhile, SA mediates various biotrophic and hemi-biotrophic pathogen resistance (Ding et al. 2022). Studies indicate that JA signaling can reduce SA addition by regulating several *NAC* TFs, including *ANAC019/055/072*. Firstly, *MYC2* promotes the transcription of these *NACs* after binding them directly to their promoters. Subsequently, the induction of SA methylation by activating these *NAC TFs* triggers benzoic acid/sa carboxyl methyltransferase 1 (*bsmt1*) expression. It inhibits SA biosynthesis by isochorismate synthase 1 (ics1) (Choudhary et al. 2021). Beyond that, *MAPK* is only one of the numerous components engaged in JA-SA signaling pathway crosstalk; *Thioredoxin (TRX) GRX* and redox regulators; *TGAs, MYC2, PDF 1.2*; and *WRKY70* (Sultana et al. 2024). When exogenous SA is present, it activates the protein *NPR1*, which in turn triggers the transcriptional activity of *WRKY70*. This activates *PR1* synthesis by binding to its promoter region and producing a defensive response. (Panpatte et al. 2020). The redox state changes SA triggers cause *TRX* to break down *NPR1* polymers into monomers. These monomers, including *GRX480*, preferentially bind *TGAs* in the nucleus. *TGAs* directly regulate *PR1*. Thus, transforming *NPR1* polymer to monomer regulates defense gene expression in two ways. GRX activation may inhibit *TGA*-mediated expression of JA response genes, including *ORA59*, promoting SA-JA antagonism (Pandey et al. 2023). *MPK4* negatively and positively controls the SA signaling pathway *GRX480* and JA signaling pathway *MYC2*. *MYC2* and its upstream *MPK4* affect plant disease resistance to necrotrophic or hemi-biotrophic infections via JA and SA signaling pathways. Activating JA-sensitive genes like *plant defensin 1.2* and *thionin 2.1* needs this regulation (Yang et al. 2019). SA activates plant defense genes early in infection. Meanwhile, infected plants produce more defense-related genes at a later stage of infection when JA is present, especially during necrotrophic or hemi-biotrophic conditions (Kalsi 2022).

**Table 2:** SAs and JAs crosstalk in stress resilience

S.No	Stress type	Biotic/ abiotic	Plant	SA-JA crosstalk interaction	TFs, genes, and responses involved in stress resilience	References
1	Fungal Stress					
	i). *Apiosporina morbosa* (Cause Black Knot)	Biotic	*Prunus salicina* and *Prunus domestica*	-	SA-dominant responses defend against biotrophic fungal infections ( second stage), reducing disease development and sustaining plant yield by SA-mediated PR1 genes.	(Shinde et al. 2024)
	ii). *Melampsora larici-populina* (Rust Fungus)	Biotic	*Populus nigra*	+	Increased SA and JA concentrations induce gene expression of *WRKY* transcription factors, *PR proteins*, phytoalexins, and anti-microbial metabolites (kaempferol, naringenin, quercetin-glycoside, rutin and flavan-3-ols)	(Ullah et al. 2022)
	iii). *Marssonina rosae* (Cause Black Spot)	Biotic	*Rosa chinensis*	-	*RcWRKY40* mediates SA-JA pathways, influencing IAA, ethylene accumulation, and the CAT antioxidant system	(Zheng et al. 2024)
	iv).* Melampsora arici-populina*	Biotic	*Populus euramericana*	+	JA-SA-common responsive genes (943) regulate the balance between growth, antioxidants, and defense responses by enhancing several metabolism pathways (phenylpropanoid biosynthesis, starch and sucrose metabolism, diterpenoid biosynthesis, and carotenoid biosynthesis).	(Luo et al. 2019)
2	Bacterial Stress					
	i). *Pseudomonas syringae pv. Actinidiae* ( Cause Kiwi Bacterial Canker)	Biotic	*Actinidia chinensis*	-	The *Psa* bacterial population in MeJA-applied plants rose 7.4-fold, but SA treatment resulted in lower *Psa* colonization (0.5-fold) compared to non-treated inoculation plants. SA enhances total carotenoids and polyphenols as defense metabolites.	(da Silva et al. 2021)
	ii). *Pectobacterium brasiliense* and *Pectobacterium atrosepticum*	Biotic	*Arabidopsis thaliana*	+	The *Exl1*-induced resistance against bacterial infection relies on SA-JA pathway interaction. PDF1.2 & AOS (JA) and (*PR1 & EDS5*) SA-JA induce response gene reduced 3.3 fold ROS accumulation in the infected plant.	(Narváez-Barragán et al. 2020)
	iii). *Ralstonia solanacearum*( Cause Bacterial Wilt)	Biotic	*Capsicum annuum*	-	Increased resistance against bacterial wilt by SA-induced immunity responses by upregulation of *CaASR1* and *CabZIP63* genes. This connection strengthens SA-dependent immunity and suppresses JA-dependent responses, providing a specific resistance against bacterial wilt.	(Huang et al. 2020)
3	Viral Stress					
	i). Tomato Yellow Leaf Curl Virus (TYLCV)	Biotic	*Solanum lycopersicum*	-	JA controls phenylpropanoid and amino acid metabolism and activates defense genes. SA seems to block defense-related processes such as flavonoid production and lipoxygenase activation.	(Wang et al. 2022b)
	ii). Rice stripe virus (RSV)	Biotic	*Oryza sativa*	+	*OsNPR1* disrupts *OsJAZ-OsMYC* to activate JA signaling. This disruption boosts antiviral immunity by activating *OsMYC2*, a critical JA signaling transcription factor. *OsNPR1* inhibits RVS Coat protein genes (*CP*).	(Zhang et al. 2023a)
4	Insect- Pest Attack					
	i). *Oebalus pugnax*	Biotic	*Oryza sativa*	-	The *OsBISAMT1* gene activates SA and JA in response to pathogens or mechanical stress infection. SA inhibits green leaf volatiles (insect attractant), such as 1-hexanol-2-ethyl (which is synthesized by JA), and enhances insect repellents (Methyl salicylate)	(Stella de Freitas et al. 2019)
	ii). *Drosophila suzukii*	Biotic	*Vitis vinifera*	+	Increase defensive secondary metabolites, including phenols, flavonoids, and tannins, minimize pest-induced damage, and make treated plants less appealing to *D. suzukii* oviposition, resulting in fewer eggs deposited	(Hussain et al. 2023)
5	Nematode & Phytoplasma					
	i).*Meloidogyne incognita* (RKN)	Biotic	*Solanum lycopersicum*	-	SA upregulates the SA-dependent defense gene (*PR1a and PR-P6*) and downregulates the JA-responsive gene (Multicystatin and Proteinase inhibitor II). SA-dependent root defenses increase nematode resistance. Jasmonate-dependent defense reduces nematode growth and reproduction during feeding. After sustained extortion, nematode egg-induced SA-dependent defenses in adult root galls increase host resistance in early parasite invasion.	(Martínez‐Medina et al. 2017)
		Biotic	*Citrullus lanatus*	+	RKN-infected plants treated with RL temporarily upregulated defense-related genes *PR1* and *WRKY70* at seven days post-inoculation. *WRKY70*, a transcription factor, integrates SA and JA signaling pathways, whereas *PR1* is connected with SA signaling.	(Yang et al. 2018b)
	ii). *Aphelenchoides besseyi* (Rice white tip nematode)	Biotic	*Oryza sativa*	+	The application of exogenous SA analog (BTH) and methyl jasmonate (MeJA) successfully induced resistance in rice plants against *A. besseyi* spatially at the flowering stage. This implies activating these hormone pathways can improve the plant's defense mechanisms.	(Xie et al. 2022)
	iii). Jujube witches’ broom disease (JWB) (Phytoplasma infection)	Biotic	*Ziziphus jujube*	. -	SA initially helps recognize pathogens and activate defenses, but switching to JA signaling with regulated ROS generation provides long-term resistance, *TIFY 6B, MAPKS, and DAGS* upregulation. This regulated hormonal interaction helps manage and mitigate JWB, allowing for resistant jujube cultivars.	(Wang et al. 2022a)
	iv). *Candidatus Phytoplasma ziziphi* (Jujube witches’ broom disease)	Biotic	*Ziziphus jujuba*	+	SA and JA activate particular *WRKY* genes that boost phytoplasma defenses. Crosstalk by WRKY TFs like *ZjWRKY42, ZjWRKY52*, and *ZjWRKY61* organizes the plant's defensive response, improving its resistance and recovery from infection.	(Fu et al. 2018)
6	Heat Stress	Abiotic	*Gracilariopsis lemaneiformis*	216 genes “ + ” and 18 genes “ -”	SA and MJ modulate photosynthesis-DAGs (cytochrome c550, and CP47 chlorophyll apoprotein), glycometabolism-DAGs (glucokinase (GK), acetyl-CoA synthetase (ACS) and phosphoglucomutase (PGM)), protein synthesis-DAGs, heat shock-DAGs (Hsp 90 & Hsp70), and signal transduction to increase heat stress tolerance.	(Wang et al. 2017)
7	Drought Stress	Abiotic	i). *Triticum aestivum*	+	Enhanced germination and water uptake, reduced water loss and proline content, and improved drought stress mitigation.	(Ilyas et al. 2017)
			ii). *Zea mays*	+	Reducing lipid peroxidation, LOX activity, and H_2_O_2_ production, increasing the accumulation of osmolytes like proline, carbohydrates, and soluble sugars, and doubling key antioxidant enzyme activity improved the plant's ability to scavenge reactive oxygen species and mitigate drought-induced oxidative damage.	(Tayyab et al. 2020)
8	Metal Stress					
	i). Ni toxicity	Abiotic	*Alyssum inflatum*	+	SA and JA treatments were able to reverse the negative impacts of Ni on plant growth, oxidative status, antioxidant enzyme (POD, CAT, GPX, APX) activities, and the accumulation of protective pigments and osmolytes	(Najafi Kakavand et al. 2019; Najafi-Kakavand et al. 2023)
	ii). Pb toxicity	Abiotic	*Zea mays*	+	SA + JA increased the uptake of essential nutrients (N, P, and K) required for plant growth and photosynthesis, as well as proline, soluble sugars, and antioxidant activity, while decreasing electrolytic leakage, MDA, and Pb concentration.	(Sofy et al. 2020)
9	Salt Stress	Abiotic	i). *Rosmarinus officinalis*	+	SA reduced salt stress's harmful effects on plant growth, photosynthetic pigment, proline, and antioxidant enzymes. Salinity, SA, and JA interaction significantly influenced protein content and plant weight.	(Nahrjoo and Sedaghathoor 2018)
			ii). *Citrus sinensis*	+	Both SA and MeJA treatments improved the expression of pathogenesis-related (*PR*) genes such as *PR1,2,3,4 &5*, plant growth, antioxidant defenses (including enzymes like POD, PAL, CAT, GSTs, APX1, CSD), and osmoregulation when plants were under salt stress.	(Mahmoud et al. 2021)

+ Shows synergistic interaction, and—shows antagonistic interaction

### SAs and JAs crosstalk in pathogen stress

Plant defense systems depend on a very dynamic and well-coordinated network of signaling networks controlling immunological responses to infections. Among the many plant defense hormones, SA and JA have crucial and mostly antagonistic functions in controlling plant responses to different pathogens. The signaling pathways in both JA and SA involve the build-up of biosynthesizing enzymes, receptors, TFs, and immune-responsive genes further down the chain. These pathways are linked and work together to fight pathogens and regulate growth. Resistance provided by biotrophic pathogens such as *Pseudomonas syringae* activates SA-dependent *PR1* expression. These infections' effectors limit JA synthesis, hence preserving SA dominance. Mutants lacking SA signaling—*npr1*—show increased sensitivity to biotrophic infections (Tyagi et al. 2022; Chan 2022). JA signaling for defense is induced by necrotrophic infections such as *Botrytis cinerea*. Pathogen penetration is limited by JA-mediated callose deposition and ROS generation. Increased JA responses in SA-deficient plants provide more resistance to necrotrophs (Marín 2023). The most recent research concluded that the interaction between SA and JA is crucial to promoting resistance to *Botryosphaeria dothidea* infection. According to this research, overexpressing *PcMYB44* increases the expression levels of key genes involved in the JA, SA, and ET signaling pathways, which improves the expression of lignin biosynthesis genes and increases lignin content in plant tissues. Lignin is an important component of the plant's defense against infections because it reinforces cell walls and makes it difficult for fungus to enter. The activation of both the SA and JA pathways, together with enhanced lignification, promotes a synergistic response that increases the plant's defense, making it more resistant to pear ring rot disease fungal (*Botryosphaeria dothidea*) pathogens (Lv et al. 2025). In a study conducted by Yu et al. (2019), it was found that *VvDOF3* may regulate the expression of genes responsible for both the SA and JA defense pathways. Additionally, the overexpression of *VvDOF3* in *Arabidopsis thaliana* increased resistance to *Golovinomyces cichoracearum*, the primary cause of powdery mildew fungal disease. The research revealed that Arabidopsis plants overexpressing *VvDOF3* exhibited increased expression of SA-responsive defense-related genes, including *PR1*. *PR1* is typically associated with resistance to biotrophic pathogens and is strongly regulated by SA signaling while antagonizing JA. Ke et al. (2019) explored the *OsEDS1*(SA-induced defense responsive gene) knockout mutant (oseds1) in rice, showing higher sensitivity to bacterial pathogens *Xanthomonas oryzae* pv. *oryzicola* (Xoo) and (Xoc) imply that *OsEDS1* is crucial in rice's immune response to bacterial infections. Interestingly, *OsEDS1* operates through the JA signaling pathway in rice, while its counterpart, *AtEDS1* in Arabidopsis, functions through the SA pathway for disease resistance. The exogenous application of JA and SA does not complement the susceptible phenotype of the *oseds1* mutant. Still, SA complements the ateds1 mutant phenotype, highlighting that SA and JA are not interchangeable in this context. Furthermore, *OsEDS1* is not required for resistance mediated by R genes in rice, whereas *AtEDS1* is essential for R gene-mediated resistance in Arabidopsis, particularly with *TIR-NB-LRR* proteins, suggesting that SA and JA interact differently with R gene-mediated immunity in these species. However, this study provides no information about the mechanism underlying the *OsEDS1*-regulated SA- and JA-related signaling pathway, which requires further study. In Arabidopsis, 3-pentanol induces an integrated defensive response by activating SA and JA defense pathways. In 2015, Song et al. (2015) discovered that 3-pentanol exposure primed the plant's immune system for increased resistance against *Pseudomonas syringae* pv. tomato DC3000 by upregulating *PR1* (related to SA signaling) and *PDF1.2* (related to JA signaling). This study emphasizes the potential of volatile compounds, such as 3-pentanol, to control SA and JA pathways, offering a novel approach for producing systemic acquired resistance (SAR) and induced systemic resistance (ISR) in crops, providing a broad-spectrum plant protection strategy. Research shows that *SlMAPK3*, a mitogen-activated protein kinase, participates in the antiviral response to *Tomato yellow leaf curl virus* (TYLCV) by regulating the expression of defense-related genes associated with both SA and JA signaling pathways. Exogenous application of SA and MeJA induced the expression of *SlMAPK3*, which, in turn, upregulated SA/JA-mediated defense genes like *PR1, PR1b/SlLapA, SlPI-I*, and *SlPI-II*. *SlMAPK3* overexpression improved resistance to TYLCV by decreasing the accumulation of ROS and increasing the activity of antioxidant enzymes (e.g., POD, SOD, CAT, and APX) (Li et al. 2017). Recent studies by Wang et al. (2022b), presented in Table 2, indicate that the interaction between SA and JA pathways is crucial for improving viral stress resistance in tomatoes (*Solanum lycopersicum*) against TYLCV. The interaction between SA and JA is crucial in enhancing viral stress resistance in tobacco plants, particularly against the *Tobacco mosaic virus* (TMV), which Yang et al. (2018a) proved in their study. This study demonstrated that the application of inhibitors of SA (1-Aminobenzotriazole, ABT) and JA (ibuprofen) diminishes the α-MMC-induced resistance to TMV, indicating that both SA and JA are required for full activation of the plant's antiviral response. SA and JA presumably interact to generate a synergistic defense response, enabling the plant to effectively manage TMV's viral impact and oxidative stress. Thus, the manipulation of SA-JA crosstalk controls the enhanced resistance to viral stress in tobacco by boosting the immunological response of the plant and thus regulating essential defense genes, including the *N*-gene correlated with TMV resistance.

Plants respond to herbivory using the phytohormone JA and its activated metabolite, JA-Ile. Plants mostly react to biotrophic diseases and sucking herbivores with SA via means of herbivore-induced plant volatiles (HIPV) synthesis (Wang et al. 2021a). We explore the dynamic relationship between SA and JA in tobacco plants' defense against herbivore offenses. Study results show that in consecutive infestations, JA accumulates in response to leaf-chewing insects, like caterpillars, increasing resistance to leaf-chewing and phloem-feeding insect assaults. On the other hand, SA primarily controls defenses against later-stage whitefly attacks. Notably, despite their well-documented hostile interaction, we found that JA and SA accumulation do not significantly affect each other's constitutive levels. The result implies that tobacco's SA/JA interaction is more complex and might be context-dependent, changing depending on various herbivore species and phases of attack rather than a simple antagonistic connection. This research data revealed that SA and JA contents exhibited antagonism only at 1 h post-whitefly infection. The SA content rose dramatically during that period, while the JA content dropped. SA content then rose constantly and peaked at 48 h; JA content remained the same as untreated plants at 48 h. These findings showed that the antagonism of JA-SA emerged in the early stage of whitefly-host interaction, not the whole time and that the whitefly infestation only raised SA content rather than JA (Liu et al. 2024).

Future research should explore how these signaling pathways integrate over time and across different environmental conditions to fine-tune plant responses to herbivore challenges. Plant-parasitic nematodes (PPNs) are a major agricultural pest, causing an estimated 80 billion USD in crop damages annually. PPNs infect many economically essential crop families (Phani et al. 2021). JA and SA play a vital role against nematodes. In *Cucumis metuliferus*, CM (resistant line), the synergistic interaction of SA and JA signaling pathways most certainly helps to coordinate a more potent defensive response against *Meloidogyne incognita*. This interaction improves the plant's recognition and response to nematode attacks, lowering the second-stage juvenile (J2) penetration and resulting in smaller giant cells and fewer nematode galls. By contrast, these pathways are less active in the susceptible cucumber line Q24, permitting deeper nematode penetration and successful development, producing the usual indications of root damage (Li et al. 2021). Table 2 presents recent novel research highlighting the complex interactions between SA and JA under various biotic stresses, including fungal, bacterial, viral, insect, pest, plant-parasitic nematode, and phytoplasma-induced damages. These studies demonstrate how SA and JA may interact antagonistically or synergistically depending on the nature of the stress and the specific plant response required. The table synthesizes key findings that illustrate how these signaling pathways are modulated by different pathogens and pests, offering valuable insights into how plants coordinate their defense mechanisms to balance the trade-offs between growth and immunity. Understanding these interactions is crucial for unraveling the complexity of plant immune responses and developing strategies to enhance crop resistance to a wide range of biotic stresses.

## SAs and JAs crosstalk in abiotic stress resilience

**Fig. 5:**
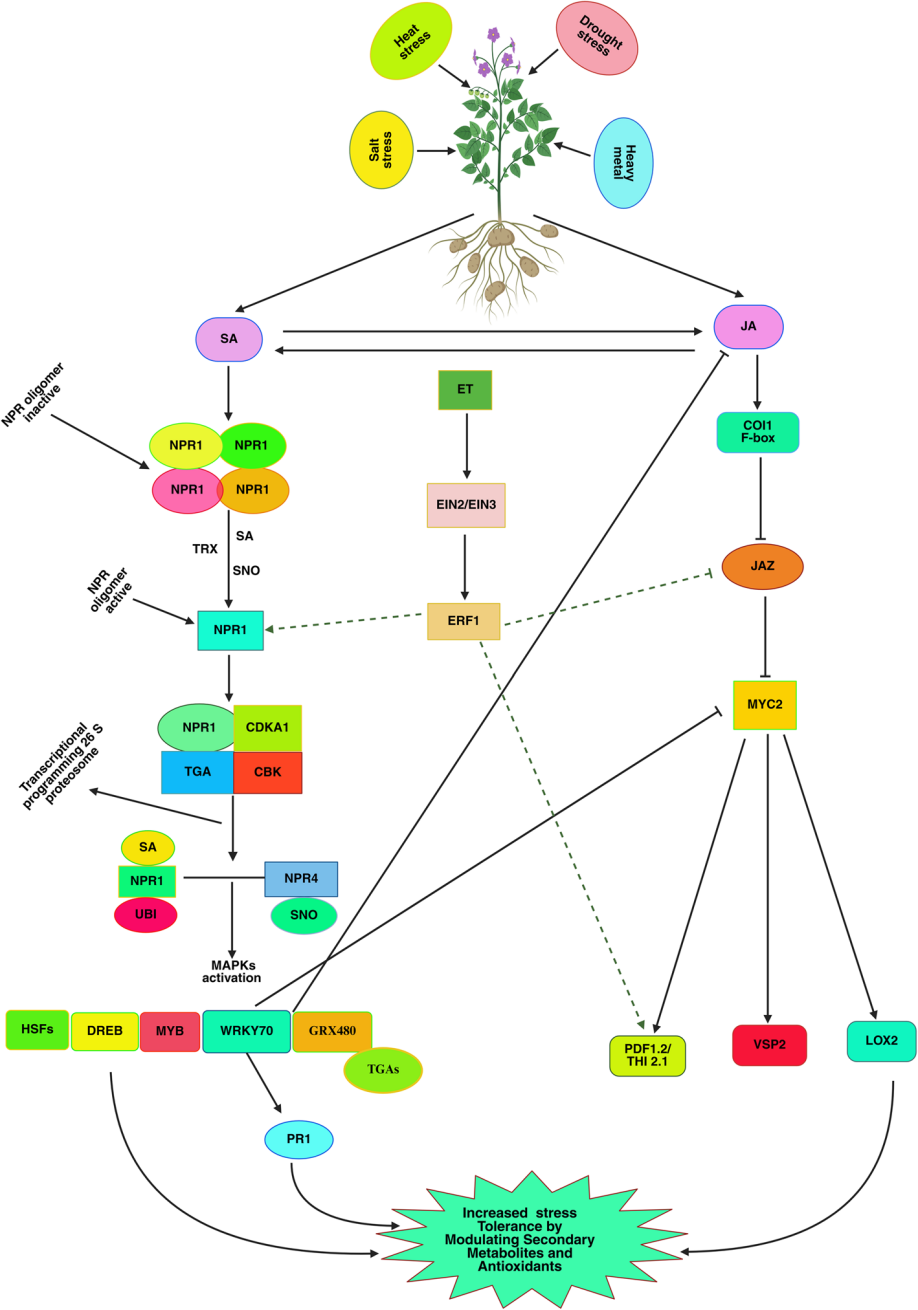
The figure highlights the critical interaction nodes between SA and JA signaling pathways under abiotic stress. This includes the involvement of transcription factors, protein kinases, and other regulatory elements. The diagram depicts various plant stress responses, including heat, salt, and drought. These stresses trigger the activation of signaling pathways, such as the NPR (Non-Expressor of Pathogenesis-Related Genes) and MAPK (Mitogen-Activated Protein Kinase) pathways. The activation of these pathways leads to the upregulation of various transcription factors and genes, including NPR1, CDKA1, TGA, CBK, UBI, SNO, and others. These transcriptional and signaling events ultimately result in the increased stress tolerance of the plant, as indicated by the "Increased Stress Tolerance" label in the diagram. The diagram also shows the involvement of various downstream effectors, such as HSFS, DREB, MYB, WRKY70, GRX480, and TGAs, contributing to the plant's stress response and adaptation. Additionally, the diagram highlights the role of specific proteins, such as JAZ, COI1 F-box, MYC2, PDF1.2/THI2.1, VSP2, and LOX2, in the plant's stress response and defense mechanisms (Created with BioRender.com)

However, SA and JA often appear antagonistic; specific scenarios provide synergistic control—especially in abiotic stress responses (such as drought, salt, and heavy metal stress), as presented in Fig. 5 (Santisree et al. 2020). Under combined stress, SA and JA can co-regulate transcription factors, including ERF (Ethylene Response Factor), which activate both the SAR and ISR pathways. This process starts with stress sensing via ROS production and calcium signaling, which triggers the synthesis of SA via the ICS and PAL pathways in plastids and cytoplasm, while JA is produced via the LOX, AOS, and OPR3 pathways in plastids and peroxisomes (Kachroo and Kachroo 2012; Roychowdhury et al. 2024, 2025). In exposure to abiotic stress, SA and JA connect on ethylene (ET) signaling, where SA stabilizes *NPR1* (*Nonexpressor of Pathogenesis-Related Genes 1*) and JA deactivates JAZ (Jasmonate ZIM-domain) repressors, enabling ERFs like ERF1, ERF5, and ERF6 to act as transcriptional integrators. ERF1 attaches to GCC-box elements in the promoters of PR (Pathogenesis-Related) genes, which turns on SAR. At the same time, it controls *PDF1.2* (*Plant Defensin 1.2*) and other JA-responsive genes, which turns on ISR (Gómez-Cadenas et al. 2014; Wu et al. 2022; Montejano-Ramírez and Valencia-Cantero 2023). This bidirectional regulation provides broad-spectrum resistance against abiotic stress by increasing stomatal closure (by SA), hardening the cell wall with lignin (via JA), and coordinating osmoprotectant accumulation (via ET) (Song et al. 2023; Lima et al. 2020; Sharma et al. 2019). Despite these achievements, there are still gaps in knowledge about how ERFs prioritize SA or JA signaling under different stress levels, the precise chromatin modifications affecting ERF-mediated gene expression, and whether further post-translational modifications tune this crosstalk for particular stress levels. SA and JA also work synergistically via abscisic acid (ABA) signaling to modulate drought and salt stress responses. The complex hormonal crosstalk network of SA, JA, ABA, and ET controls plant responses to drought and salt stress while maintaining growth, defense, and stress tolerance (Riemann et al. 2015). Under water scarcity or salt exposure, ABA accumulates in guard cells and induces stomatal closure via ABA-responsive PYR/PYL/RCAR receptors, which block PP2C phosphatases, activating SnRK2 kinases that phosphorylate and increase ABA-responsive genes (e.g., *RD29A*, *NCED3*) (Hewage et al. 2020). The transcription factor *MYC2* interacts with ABA-responsive elements (ABREs) to increase drought-adapted gene expression. Through its central regulator, *EIN3*, ET signaling modulates ABA and JA responses in this network by suppressing ABA-induced stomatal closure, enabling appropriate gas exchange during moderate drought. ET interacts with JA to increase drought tolerance by encouraging antioxidant enzyme expression (SOD, CAT) and osmolyte accumulation during severe stress, respectively (Muhammad Aslam et al. 2022; Ullah et al. 2018). However, SA acts a dual function: at low concentrations, it activates *WRKY* and *NAC* transcription factors, which regulate osmoprotectant biosynthesis (proline, glycine betaine), thereby synergizing with ABA and JA. However, it antagonizes ABA at high concentrations by interfering with *SnRK2* phosphorylation, thereby delaying stomatal closure (Samanta and Roychoudhury 2025). The delicate equilibrium between these hormonal pathways determines whether plants prioritize growth (ABA-JA-ET synergy) or long-term stress defense (SA-JA antagonism with ABA).

Plants may be more resistant to salt and drought stress by increasing their synthesis of SA and JA, either genetically or biochemically. Recent research has shown that low concentrations of SA and JA work together to improve abiotic stress tolerance in plants. A study conducted on soybeans demonstrated that the foliar application of SA (1 mM) in conjunction with JA (0.5 mM) significantly increased the H^+^-ATPase hydrolytic activity of the tonoplast and the ATP content in root cells under sustained NaCl-induced salinity stress (4 dS m^−1^). The SA + JA treatment improved root development, enhanced cation absorption, increased the chlorophyll content index (CCI), and increased relative water content, total plant biomass (10%), and overall grain production (17%) at different salinity levels. The findings indicate that the combined application of SA and JA is more effective in mitigating the adverse effects of salinity on soybean growth and productivity compared to individual treatments with these phytohormones (Ghassemi-Golezani and Farhangi-Abriz 2018). It is revealed that foliar application of JA and SA improves water content and enhances leaf area index and grain yield in Safflower. Under salinity stress conditions, the presence of JA and SA led to a notable rise in catalase (CAT) activity while causing a substantial reduction in ROS generation and levels of lipid peroxidation in safflower (*Carthamus tinctorius* L.) plants (Lotfi et al. 2024). Mahmoud et al. (2021) more recently demonstrated how well exogenous MeJA and SA improved photosynthetic pigment content (Chl) and antioxidant enzyme activity (cytosolic GSTs, SOD, APX1, POD, CAT, and PAL) in *Citrus sinensis* plants to combat salt stress. By adding doses with both the phytohormones to control salt-induced damage, the authors also noted changes in the expression of pathogenesis-related genes (*PR1, PR3, PR4*, and *PR5*), the SOS family of genes (*SOS1, SOS2,* and *SOS3*), *WRKY* genes (*WRKY33* and *WRKY70*), and aquaporin genes [tonoplast intrinsic proteins (*TIP4;1*) and plasma membrane intrinsic proteins (*PIP1;1, PIP2;3*)]. Recently, MeJA and SA in *Ziziphus jujuba* plants have been shown to control a transcription factor, *ZjWRKY18*, which participates in triterpenoid biosynthesis and also performs a crucial role in salt stress resistance (Wen et al. 2023).

The combined seed and foliar pre-treatments of MeJA + SA demonstrated a synergistic effect, lower drought stress-induced oxidative damage by reducing lipid peroxidation, LOX activity, and H_2_O_2_ production and maintenance of plant water status through increasing the accumulation of osmolytes like proline, carbohydrates, and soluble sugars, and doubling key antioxidant enzyme activity improved the plant's ability to scavenge reactive oxygen species in maize (*Zea mays*) seedlings (Tayyab et al. 2020). A recent study by Mohi-Ud-Din et al. (2021) found that the combined application of SA and MeJA enhances drought stress tolerance in French bean plants by reducing oxidative stress markers such as superoxide anion (O2^•−^), H_2_O_2_, MDA, and lipoxygenase (LOX) activity. This reduction indicates better control over oxidative damage. The treatment also increases the activities of key antioxidant enzymes— SOD, CAT, POD, GPX, and glutathione-S-transferase (GST)—which neutralize ROS and protect the plant. Additionally, the combination boosts enzymes in the ascorbate–glutathione cycle, helping maintain redox homeostasis under drought stress in *Phaseolus vulgaris*. A comparative transcriptome profiling using RNA-seq revealed that JA synergistically regulated 216 genes and 18 genes were antagonistically regulated by SA in response to heat stress mitigation. This experiment demonstrated that multiple applications of MeJA and SA elicited a significant response, with their synergistic effects serving as the primary mechanism to mitigate heat stress in the economically substantial *Gracilariopsis lemaneiformis*. This is accomplished through the modulation of photosynthesis, glycometabolism, protein synthesis, heat shock responses, and signal transduction, thereby enhancing heat stress tolerance (Wang et al. 2017). Excessive levels of heavy metals like mercury (Hg), lead (Pb), copper (Cu), and cadmium (Cd) can significantly impair the growth and development of plants. These harmful metals, whether derived from industrial waste materials or the natural environment, pose severe risks to the flourishing of plant life (Khatun et al. 2022). Combining jasmonic and salicylic acid treatment elevated the plant weight and mitigated Ni oxidative effects by reducing H_2_O_2_ concentration. SA and JA also increased SOD, CAT, POD, and APX in *Acisoma inflatum* (Najafi Kakavand et al. 2019; Najafi- Kakavand et al. 2023). The combined SA + JA treatment under Pb stress reduces the accumulation of Pb in maize plants, lowering membrane degradation caused by oxidative stress-induced ROS production. Also, it enhances photosynthesis-related activity, peroxidase, mineral nutrients, superoxide dismutase, phenol, ascorbic acid, catalase, glutathione, and total soluble sugar production (Sofy et al. 2020). In summary, the synthesis, sensitivity, and supplementation of SA and JA enhance a plant's ability to cope with salt and drought stress by modulating ionic transport, promoting water retention strategies, activating antioxidant defenses, and altering gene expression profiles to foster stress resilience. This integrated response helps maintain ionic and water balance, which is essential for plant survival under adverse environmental conditions.

## Engineering stress-resistant crops through SA-JA crosstalk manipulation

The severity and specificity of the plant's defensive systems depend on the SA and JA signaling balance. When these pathways are not appropriately regulated, plants may experience excessive growth inhibition, lower yields, or excessive synthesis of defense-related metabolites, which may be harmful in specific environments (Chen et al. 2022). Therefore, it is crucial to understand the precise molecular mechanisms behind SA-JA crosstalk to develop strategies that improve crop resilience without compromising productivity. Researchers may change agricultural pathways using breeding and biotechnological strategies by understanding how these two hormones interact. This might enhance plant resilience to biotic and abiotic stressors without affecting yield and production. Traditional breeding methods may select plant varieties with differences in natural SA and JA signaling systems. JA-responsive crops might be engineered to fight insect pests and diseases. Selectively breeding for increased SA accumulation may enhance fungal and bacterial resistance. Breeders may design cultivars with optimum growth and resistance by finding specific SA-JA signaling genetic loci (Rachappanavar et al. 2022).

Biotechnological ways of manipulating SA and JA signals in plants, such as genetic engineering and CRISPR/Cas9 gene editing, are more specific. For instance, the overexpression of essential proteins and genes in the JA biosynthesis pathway or the suppression of specific SA-responsive genes could enhance resistance to herbivory and pathogen attack (Bigini et al. 2021). Liu et al. (2023) use CRISPR/Cas9-mediated gene editing to boost plant disease resistance. This research of rice *OsS5H* genes emphasizes the function of SA 5-hydroxylase in controlling SA levels since overexpression decreases SA concentration, making plants vulnerable to *Xanthomonas oryzae* pv. *oryzae* (Xoo). The CRISPR/Cas9-generated *oss5h1oss5h2oss5h3* triple mutants, deficient in SA 5-hydroxylation ability, accumulate SA and are more pathogen-resistant. This improved resistance is coupled with increased expression of critical defense genes- *OsWRKY45* and *PR* genes and an amplified ROS burst after pathogen-associated molecular pattern (flg22) detection (Liu et al. 2023).

A recent study has shown that *VvWRKY5*, a *WRKY* IIe subfamily transcription factor, enhances grapevine resistance to *Coniella diplodiella*-caused white rot. Pathogen infection and JA treatment dramatically upregulate *VvWRKY5*, demonstrating its role in JA-mediated defensive responses. Functional investigations in grape and Arabidopsis show that *VvWRKY5* regulates essential factors of JA signaling pathway components to control resistance favorably. *VvWRKY5* directly represses *VvJAZ2* and activates *VvMYC2*, a JA signaling activator. Significantly, *VvWRKY5* physically interacts with *VvJAZ2*, fine-tuning transcriptional control. JA biosynthesis and signaling are enhanced by this dual mechanism, defending against white rot. These findings demonstrate a clear genetic target—*VvWRKY5*—for JA pathway enhancement via gene editing or transgenic approaches to improve disease resistance in grapevine (Zhang et al. 2023b).

Typically antagonistic, the SA and JA defensive routes imply that activation of one may inhibit the other. This adversarial relationship challenges obtaining broad-spectrum resistance, as enhancing one pathway might compromise the other. In the instance of *Pseudomonas syringae* pv. tomato (PtoDC3000), the bacterium, generates coronatine (COR), a chemical that increases stomatal opening, therefore facilitating bacterial colonization of the plant. In Arabidopsis, COR's action is mediated by the COR co-receptor *AtJAZ2*; tomato revealed a comparable functional ortholog *(SlJAZ2)*. *SlJAZ2* is a key factor in stomatal response to COR as it accumulates in stomatal guard cells. Using CRISpen/Cas9, the researchers genetically altered the *SlJAZ2* protein to produce a dominant repressor variant of *SlJAZ2 (SlJAZ2Δjas)*, which lacks the C-terminal Jas domain, therefore overcoming the trade-off between resistance to biotrophic and necrotrophic diseases. *SlJAZ2Δjas* prevents the stomatal reopening brought on by COR without compromising the plant's resilience to necrotrophic diseases like Botrytis cinerea (which produces gray mold) (Ortigosa et al. 2019). Essentially uncoupling the antagonism between SA and JA pathways, especially at the stomata, this genetic change helps tomato plants retain resistance to bacterial diseases without impairing their defense against fungal pathogens and growth. Li et al. (2015) study demonstrates that the overexpression of *SpWRKY1* in tobacco resulted in enhanced expression of SA- and JA-associated genes, such as *NtPR1, NtPR2, NtPR4, NtPR5*, and *NtPDF1.2* (SA-related) and defense-related genes like *NtPOD, NtSOD, NtPAL, NtLEA5, NtP5CS*, and *NtNCED1*. This gene expression, particularly the SA- and JA-associated genes, suggests that the balance between SA and JA signaling pathways can synergistically regulate the plant's defense mechanisms for salt and drought stress tolerance; this interaction likely leads to increased antioxidant enzyme activities (e.g., peroxidase (POD), superoxide dismutase (SOD)) that help mitigate oxidative stress during salt and drought conditions, enhanced chlorophyll content and photosynthetic rate, which contribute to improved plant growth and survival under adverse conditions. Higher stomatal conductance ensures better water regulation under stress conditions.

## Current knowledge gaps and future directions

SA and JA functions in plant stress resistance have been better understood. However, some critical issues remain unanswered, impeding their comprehension of interaction. For example, SA-JA cross-talk regulates responses to various biotic and abiotic stressors; the molecular mechanisms of their dynamic interplay remain elusive. In particular, SA and JA signaling pathway timing and sequencing during stress events are unknown. The effects of light, temperature, and soil composition on this interaction have not been extensively studied. Despite these gaps, unanswered issues influence existing knowledge and provide potential research possibilities.

### Key questions

- The temporal regulation of the SA-JA interaction and its impact on stress tolerance is unclear.
- Scarcity of information on species-specific modulation of SA-JA interaction in stress resilience.
- How do SA and JA crosstalk contribute to long-term plant memory of environmental stress?
- The role of SA-JA-induced epigenetic changes in modulating stress tolerance is primarily unexplored.
- How does ET resolve SA-JA's inherent antagonism in stress?

Future research should focus on these unresolved questions and the signaling networks involved in the interaction between SA and JA. A deeper understanding of this cross-talk could provide valuable insights into improving the adaptive capacity of plants. Specific areas for further investigation include manipulating the activity of enzymes involved in SA and JA biosynthesis, such as ICS1 (for SA) and AOC (for JA), which may result in crops with higher resistance to specific stressors. For example, improving SA biosynthesis while lowering JA production could make crops more pathogen-resistant without suffering detrimental consequences for growth. Another method implies altering the expression of critical receptors that detect SA and JA, including NPR1 (for SA) and COI1 (for JA). Plants may show an ideal balance between defense and growth responses by selectively increasing or decreasing the sensitivity of these receptors, strengthening their capacity to resist many stresses simultaneously. This can shift the hormonal equilibrium in favor of increased stress resilience. Also, it has enormous potential for commercial crop protection through PGR spray during stress, which is where future research is needed.

## Conclusion

The interplay between SA and JA facilitates stress tolerance in plants. This interaction forms a complex signaling pathway network that boosts plants' capacity to withstand biotic and abiotic stressors. These phytohormones interact antagonistically and synergistically to enable a well-regulated system that allows plants to establish suitable defenses without sacrificing growth and development. Progress in comprehending these relationships has clarified crucial parts of plant biology and created opportunities for cultivating crops with improved stress resilience using biotechnological and breeding methods. The further study focused on understanding the intricacies of salicylate and jasmonate signaling integration, which holds the potential to enhance our abilities to improve agricultural production and sustainability in response to increasing environmental challenges.

## Data Availability

NA.

## Abbreviations

SA: Salicylates
JA: Jasmonates
SAR: Systemic acquired resistance
ROS: Reactive oxygen species
PGR: Plant growth regulators
ABA: Abscisic acid
CK: Cytokinin
ET: Ethylene
BRs: Brassinosteroids
JA-Ile: Jasmonic acid isoleucine
MeJA: Methyl Jasmonate
PPN: Plant parasitic nematodes
